# Bone Marrow Mononuclear Cells Up-Regulate Toll-Like Receptor Expression and Produce Inflammatory Mediators in Response to Cigarette Smoke Extract

**DOI:** 10.1371/journal.pone.0021173

**Published:** 2011-06-29

**Authors:** Junmin Zhou, Erika A. Eksioglu, Nicole R. Fortenbery, Xianghong Chen, Huaquan Wang, Pearlie K. Epling-Burnette, Julie Y. Djeu, Sheng Wei

**Affiliations:** 1 Department of Immunology, H. Lee Moffitt Cancer Center and Research Institute, Tampa, Florida, United States of America; 2 Department of Hematology, General Hospital, Tianjin Medical University, Tianjin, China; University of Southern California, United States of America

## Abstract

Several reports link cigarette smoking with leukemia. However, the effects of cigarette smoke extract (CSE) on bone marrow hematopoiesis remain unknown. The objective of this study was to elucidate the direct effects of cigarette smoke on human bone marrow hematopoiesis and characterize the inflammatory process known to result from cigarette smoking. Bone marrow mononuclear cells (BMCs) from healthy individuals when exposed to CSE had significantly diminished CFU-E, BFU-E and CFU-GM. We found increased nuclear translocation of the NF-κB p65 subunit and, independently, enhanced activation of AKT and ERK1/2. Exposure of BMCs to CSE induced IL-8 and TGF-β1 production, which was dependent on NF-κB and ERK1/2, but not on AKT. CSE treatment had no effect on the release of TNF-α, IL-10, or VEGF. Finally, CSE also had a significant induction of TLR2, TLR3 and TLR4, out of which, the up-regulation of TLR2 and TLR3 was found to be dependent on ERK1/2 and NF-κB activation, but not AKT. These results indicate that CSE profoundly inhibits the growth of erythroid and granulocyte-macrophage progenitors in the bone marrow. Further, CSE modulates NF-κB- and ERK1/2-dependent responses, suggesting that cigarette smoking may impair bone marrow hematopoiesis in vivo as well as induce inflammation, two processes that proceed malignant transformation.

## Introduction

Cigarette smoking is a substantial contributor to the pathogenesis of many diseases, including cancer, predisposition to infections as well as a contributing factor to the failure of therapeutic modalities, such as HSCT transplants [1], [2], [3], [4], [5]. Numerous studies have suggested a strong link between exposure to cigarette smoke (CS) and an increase in the risk of developing myeloid leukemia in adults, as well as the association between parental smoking and childhood lymphomas and leukemias [6], [7]. Tobacco use may result in an imbalance of the hematopoietic system, such as changes in the erythrocyte-leukocyte ratio and the composition of mature leukocytes in the peripheral blood [8]. Furthermore, recent studies have demonstrated the ability of bone marrow-derived stem cells to respond to induced epithelial repair and are also linked with cigarette smoke-induced lung cancer [9]. Interestingly, apart from these observations, the mechanisms by which smoking impairs the hematopoietic system are not well understood.

It has been reported that cigarette smoke-induced inflammation and impairment in humans is associated with bone marrow stimulation and an accelerated release of leukocytes into the circulation [10]. However, while some of the mechanisms by which cigarette smoke and CSE causes functional impairment have been studied in PBMC and non-immune cells, the direct behavior of such pathways in primary bone marrow cells remain poorly understood [11].

Toll-like receptors (TLRs) are key innate immune receptors involved in initiating the recognition of pathogens by the immune system and in facilitating the myeloid replenishment/recruitment by HSCs in response to inflammation [12], [13], [14]. Previous studies have shown that CSE can up-regulate the expression of TLR4 on human macrophages, neutrophils, and bronchial epithelial cells; TLR2 in CS–exposed mice, smokers and patients with COPD; and TLR3 in adenocarcinoma cells [12], [15], [16], [17]. The activation of TLRs promotes the activation of signaling pathways, such as phospho inositol-3 kinase (PI3K)/AKT, nuclear factor-κB (NF-κB) and extracellular signal-regulated kinase 1/2 (ERK1/2), which lead to the production of pro-inflammatory cytokines and chemokines important for immune responses [15], [18], [19]. Among these, NF-κB has been reported to play an important role in mediating cell survival and the up-regulation of many cytokines and pro-inflammatory mediators essential to the host, and ERK1/2 has been reported to mediate transcription of proteases and cytokines in response to a variety of stimuli, including CS [20]. Furthermore, a number of studies have demonstrated that NF-κB and MAPK signaling pathways become activated in response to CS [21], [22]. Therefore, several signaling pathways may be involved in modulating BMCs and HSCs survival in response to cigarette smoke-induced immunosuppressive properties.

In this study, the impact of CSE on bone marrow hematopoiesis, expression and signaling of TLR2, TLR3, and TLR4 in bone marrow mononuclear cells (BMCs) from healthy volunteers was investigated.

## Results

### Cigarette Smoke Extract Reduced the Colony Formation of BMCs

**Figure 1:**
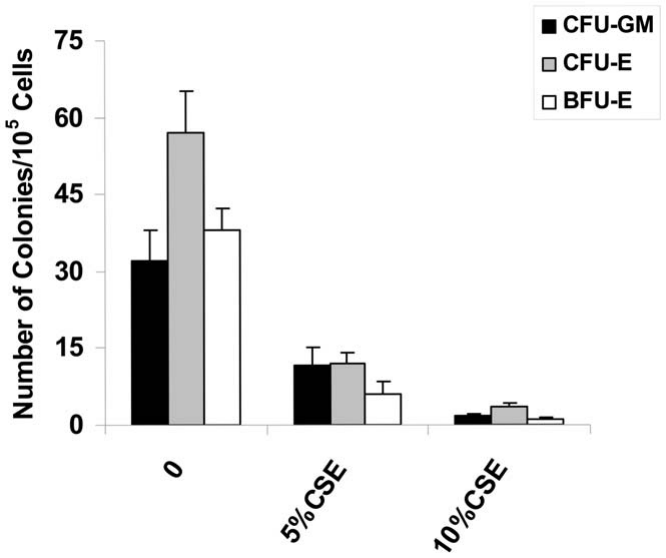
Effects of CSE on the clonal growth of normal human CFU-GM, BFU-E and CFU-E in BMCs. Bone marrow mononuclear cells from healthy volunteers were treated with different concentrations of CSE for 24 h, then the BMCs were harvested and grown in methylcellulose medium with cytokines as described in Materials and methods. At the end of the 24 h, the number of colonies per well was determined. Each point represents the mean results of three normal individuals, and each experimental point represents duplicate plates. Results are expressed as mean value ± SD (* P<0.001).

Examination of colony formation following exposure to various agents is a sensitive indicator of toxicity and as mentioned earlier, human BM-derived progenitors might be the most applicable to the toxicology of human CS exposure [23]. Therefore, our primary goal was to determine the effects of CSE on BM hematopoiesis. We did this by studying a number of different types of progenitor cell colonies formed (BFU-E, CFU-E and CFU-GM) by BMCs after exposure to different concentrations of CSE. BMCs exposed to CSE had a profound decrease in the numbers of CFU-U, BFU-E, and CFU-GM scores when compared to control treated cells (media alone). Even the lowest concentration (5% CSE) showed a significant inhibitory effect on the growth of erythroid progenitors (CFU-E and BFU-E) and granulocyte-macrophage progenitors (CFU-GM) (Fig. 1), demonstrating that progenitor cells are highly sensitive to the hematotoxic effects of CSE.

### Cigarette Smoke Extract Induces Nuclear Translocation of NF-κB and Activation of PI3K/AKT and ERK1/2 in BMCs

**Figure 2:**
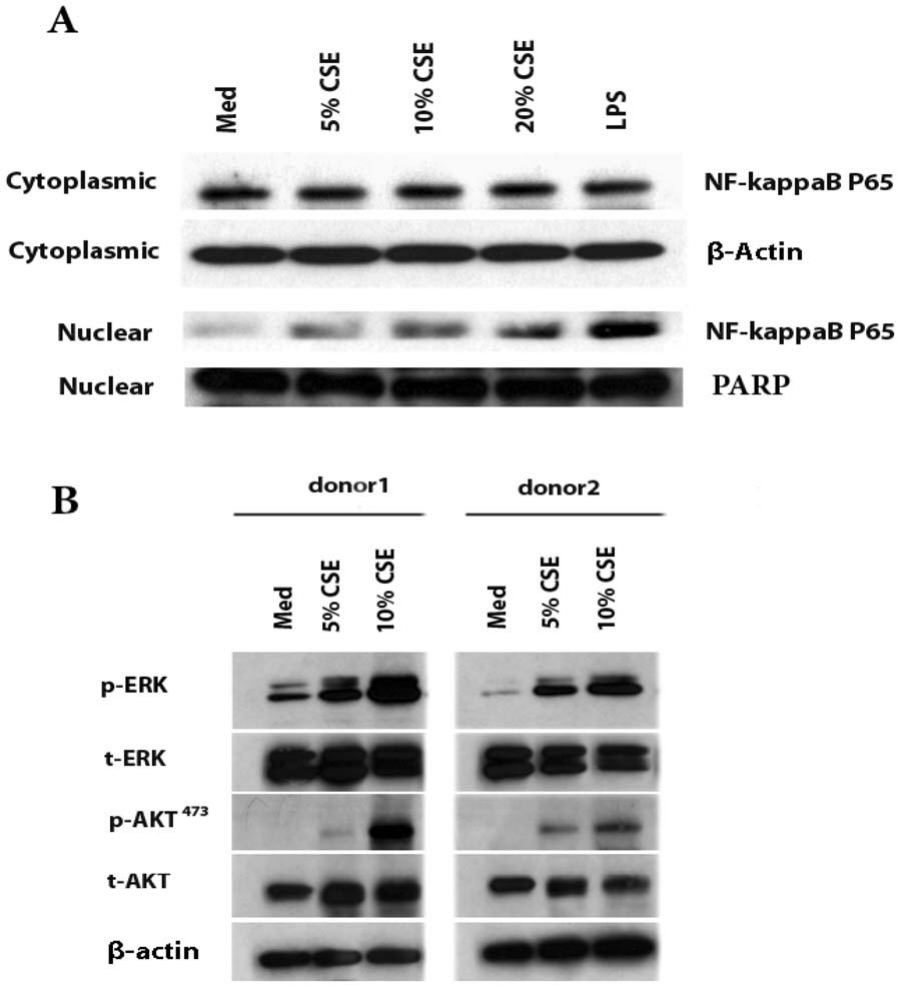
Cigarette smoke extract (CSE) induced the activation of NF-κB, AKT and ERK1/2 in BMCs. (A) BMCs were incubated with media, 5% CSE, 10% CSE, 20% CSE or 1 µg/ml LPS for 24 h, then cells were harvested and the cytoplasmic and nuclear protein fractions were separated using Pierce Nuclear and Cytoplasmic Extraction Reagent Kit (NE-PER). 30 µg of each, cytoplasmic and nuclear extracts, was analyzed by 10% SDS-PAGE and Western blotted using NF-κB p65 antibody. For loading control of cytoplasmic protein, the membrane was stripped and immunoblotted for β-actin antibody; for loading control of nuclear protein, the membrane was blotted with anti-PARP antibody. Three independent experiments were done, but one representative experiment is shown. (B) BMCs from donor 1 and donor 2 were incubated with control media, 5% CSE or 10% CSE for 6 h at which point cells were harvested and lysed. Cell lysates were separated by SDS-PAGE and immunoblotted with an anti-p-ERK antibody or anti-p-AKT (ser473) antibody, and the membranes were subsequently stripped and reprobed with anti-ERK, anti-AKT or β-actin antibodies.

NF-κB belongs to a family of transcription factors that regulates many cellular processes such as inflammation, immune activation, cell proliferation, and apoptosis [24], [25]. In most cells, NF-κB factors are stored in an inactive state in the cytoplasm until activating signals induce nuclear translocation [24], [26], [27]. Importantly, this factor has been previously shown to be activated in response to CSE exposure. Therefore, we aimed to determine whether exposure of BMCs to CSE could also result in activation of NF-κB. We treated BMCs with different concentrations of CSE for 24 hours and immunoblotted the cytoplasmic and nuclear portions for the NF-κB subunit, p65. We observed that CSE induced nuclear translocation of p65 in a dose-dependent manner (Fig. 2A). To investigate whether CSE could stimulate the activation of other pathways, BMCs were treated with 5% or 10% CSE for 6 h and CSE-mediated activation of AKT and ERK was then determined by Western Blot analysis looking at the phosphorylation status of AKT and ERK. As shown in figure 2B, we also see increased activation in response to CSE.

### IκBα Phosphorylation by CSE Is Independent of the Activation of PI3K/AKT and ERK1/2 in BMCs

**Figure 3:**
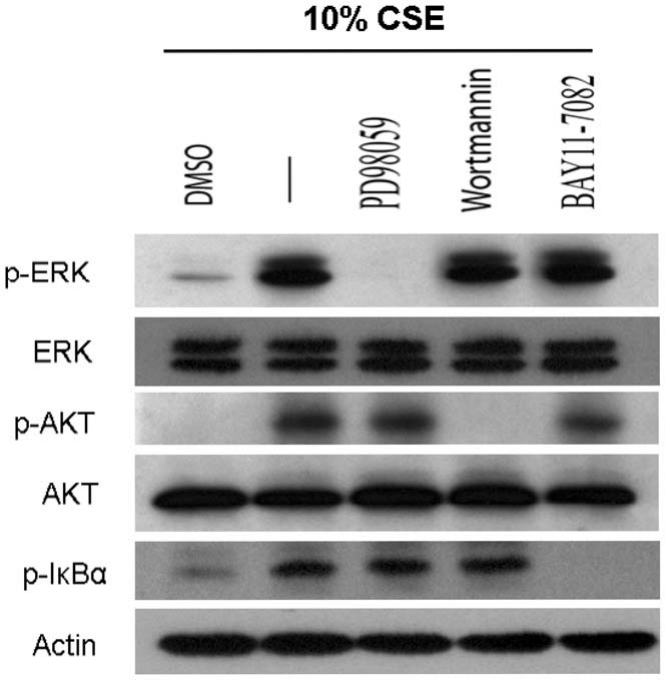
CSE-induced NF-κB activation is independent of the activation of PI3K/AKT and ERK1/2 in BMCs. BMCs were pretreated with PD98059 (10 µM), BAY11-7085 (5 µM), or Wortmannin (100 nM) for 60 min, and were subsequently exposed to 10% CSE for 6 h then cells were harvested and lysed. Cell lysates were separated by SDS-PAGE and immunoblotted with an anti-p-ERK antibody, anti-p-AKT (Ser473) or anti-p-IκBα (Ser32) antibody. The membranes were then stripped and reprobed with anti-ERK, anti-AKT or β-actin antibodies.

The PI3K/AKT signaling pathway has been shown to activate NF-κB [28], [29]. As shown above, CSE can activate PI3K/AKT signaling, therefore, we reasoned that this pathway might also be responsible for activating NF-κB in BMCs. To test this, BMCs were pretreated with Wortmannin (PI3K inhibitor) and then stimulated with CSE. In an unactivate state, NF-κB is sequestered in the cytoplasm by the action of an inhibitory protein IκBα, Upon cellular activation, IκBα becomes phosphorylated and subsequently degraded, and NF-κB is released and free to translocate into the nucleus where it can then regulate the transcription its target genes. [30]. Therefore we tested if there was a link between the phosphorylation of IκBα and the PI3K pathway in CSE-induced activation of NF-κB. We examine IκBα phosphorylation after treatment with CSE, with and without inhibition of PI3K. We found that while CSE-mediated phosphorylation of AKT was effectively ablated by PI3K inhibition, CSE-induced IκBα phosphorylation in BMCs was not (Fig.3). These data demonstrate that PI3K/AKT signaling does not lie upstream of NF-κB in CSE-pathogenesis and is not responsible for the CSE-induced NF-κB activity seen in BMCs.

We showed earlier that MAPK in BMCs (represented by ERK1/2) was also hyperactive in response to CSE and numerous reports have shown that CSE can activate the MAPK signaling pathway [20], [21], [22]. Therefore, we assessed the impact of ERK activation on CSE-induced NF-κB activation in BMCs. Cells were pretreated with 10 µM of the MAPK inhibitor, PD098059, for 1 h and then stimulated with CSE for 6 h, as we did for PI3K with Wortmaninn. We were able to block CSE-mediated ERK1/2 activation using PD098059, but there was no significant change in CSE-induced IκBα phosphorylation, indicating that CSE has a direct effect on NF-κB activation in BMCs.

### CSE Induces TLR Expression on BMCs

**Figure 4:**
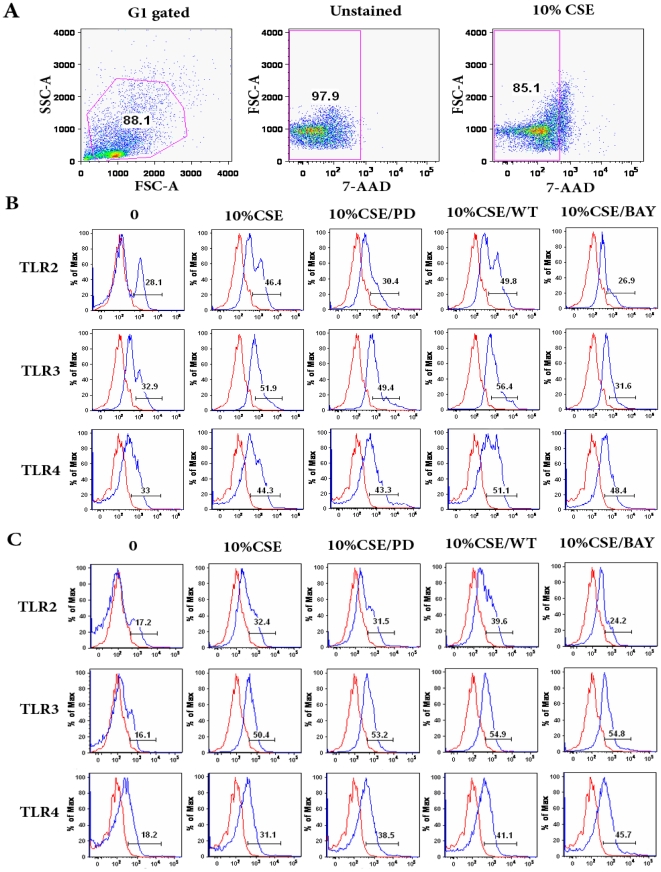
CSE-induced TLR2 and TLR3 expression in BMCs may be in part mediated through activation of ERK1/2 and NF-κB signaling cascades. BMCs were pretreated with PD98059 (10 µM), BAY11-7085 (5 µM), or Wortmannin (100 nM) for 60 min, and then exposed to 10% CSE for 24 h, after which the cells were harvested and used for flow cytometric analysis. (A) BMCs were gated according to forward scatter (FSC) and side scatter (SSC) profiles. For cell surface staining, 7-AAD negative cells (live cells) were gated for TLRs expression analysis. Histograms show representative fluorescence intensities of TLRs; BMCs were labeled with anti-TLR2-PE, anti-TLR3-PE or anti-TLR4-APC antibodies (blue line histogram) or appropriate isotype control antibodies (red line histogram). (B) For intracellular TLR2, TLR3 or TLR4 staining, cells were washed and then fixed and permeabilized. (C) For cell surface expression of TLR2, TLR3 or TLR4, the cells were incubated with anti-TLR2-PE, anti-TLR3-PE or anti-TLR4-APC antibodies for 30 min in the dark on ice, washed and resuspended in PBS. The experiment is a representation of three independent experiments.

TLR4 has been implicated in the activation of AKT and MAPK and activating signals emanating from TLRs are well known to induce activation of NF-κB in antigen-presenting cells, which results in the production of several pro-inflammatory cytokines [31], [32]. To explore whether CSE is able to affect the expression of TLRs, BMCs were stimulated with 10% CSE, and then evaluated for their expression of TLR2, TLR3, and TLR4. We found that BMCs exposed to 10% CSE not only increased intracellular expression of TLR2, TLR3, and TLR4 (Fig 4B), but we also saw a dramatic increase in the surface expression of these TLRs (Fig 4C). Up to this point we have demonstrated that CSE seems to target the direct activation of NF-κB and hence we wanted to understand if the activation is linked to the up-regulation of TLRs. Pretreatment of BMCs with the NF-κB inhibitor, BAY11-7085 (5 µM), decreased the intracellular expression of TLR2 and TLR3, as well as surface expression of TLR2 (Fig 4B, C), demonstrating that CSE-induced NF-κB activation is responsible for the up-regulation of these specific TLRs. Further, ERK1/2 inhibition also leads to decreased intracellular expression of TLR2, although to a lesser extent. However, PI3K/AKT inhibition with wortmannin had no effect on TLR expression suggesting that this last pathway is not directly involved in the regulation of TLR expression after CSE exposure. Thus, exposing BMCs to CSE results in increased TLR expression with the expression of TLR2 and TLR3 being at least partially mediated through activation of ERK1/2 and NF-κB.

### CSE Has No Effect on TNF-α, VEGF and IL-10 Production by BMCs

**Figure 5:**
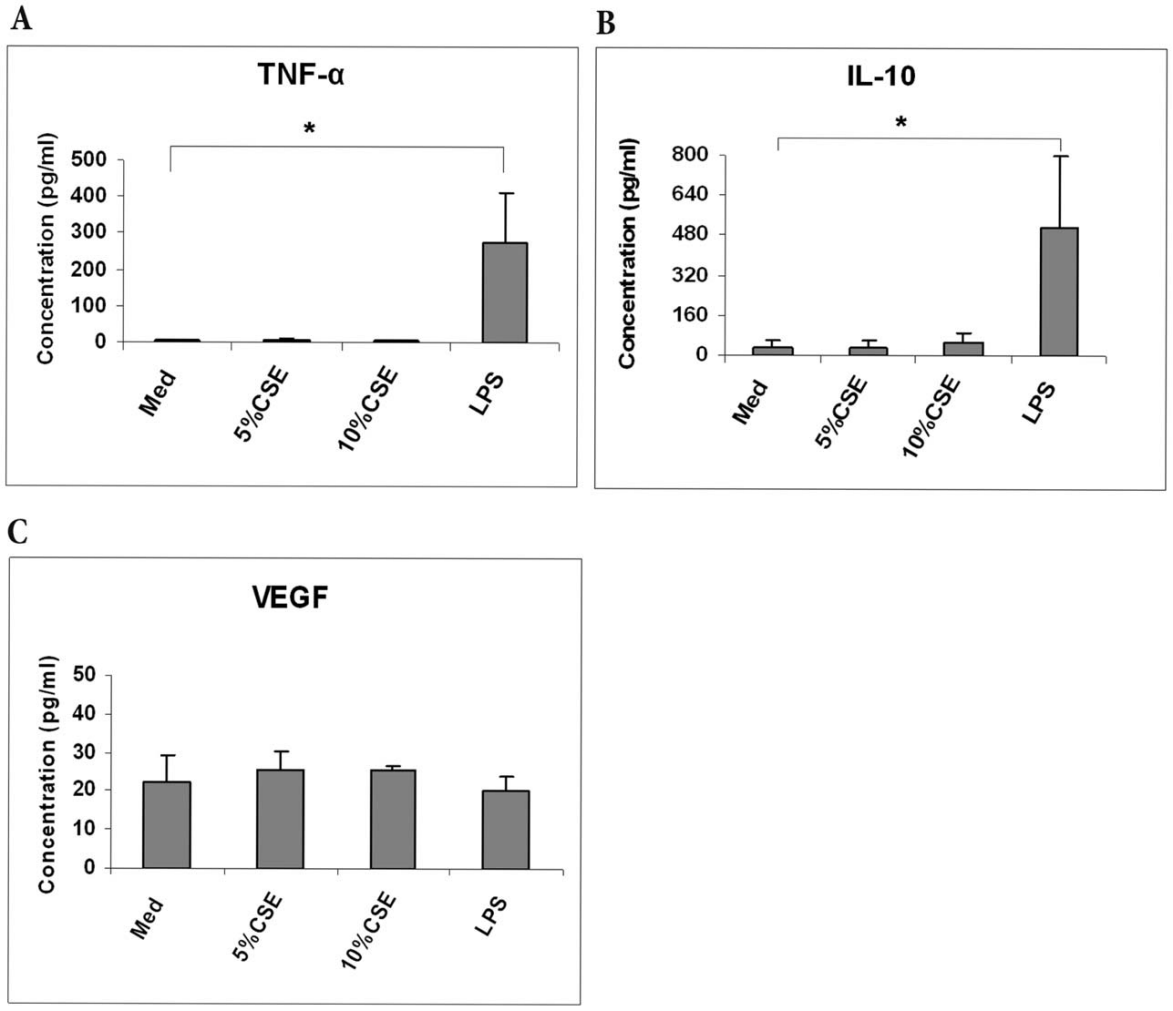
CSE had no effect on IL-10, TNF-α, and VEGF production. BMCs were incubated with media, 5% CSE, 10% CSE or 1 µg/ml LPS for 24 h, and the supernants were harvested and analyzed for the presence of IL-10, TNF-α and VEGF by ELISA. Results are expressed as mean value ± SD of five different normal donors. (* P<0.001).

Proliferation, differentiation, and self-renewal of bone marrow hematopoietic cells are in part mediated by soluble factors, including cytokines and chemokines [8]. However, the result of cigarette smoke exposure on the production and release of inflammatory mediators remains unclear. In the present study we investigated whether Tumor necrosis factor-α (TNF-α), vascular endothelial growth factor (VEGF) and, interleukin-10 (IL-10) production by BMCs is modulated by CSE. BMCs were incubated with control medium, 5% CSE, 10% CSE or 1 µg/ml of LPS for 24 h, and then the supernatants were collected and the release of TNF- α (Fig. 5A), IL-10 (Fig 5B) and VEGF (Fig. 5C) was measured by ELISA. We found that CSE did not induce the production of TNF-α, IL-10, or VEGF in BMCs.

### Effects of CSE on IL-8 Release

**Figure 6:**
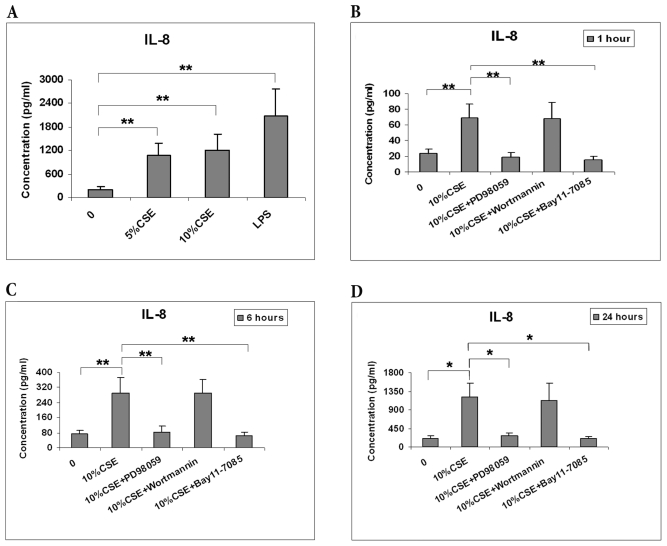
Effects of ERK1/2, AKT and NF-κB signaling pathway on CSE-induced IL-8 release. A, BMCs were incubated with media, 5% CSE, 10% CSE, or 1 µg/ml LPS for 24 h, and cell supernatants were tested for IL-8. B, BMCs were pretreated with PD98059 (10 µM), BAY11-7085 (5 µM), or Wortmannin (100 nM) for 60 min, and subsequently were exposed to 10% CSE for 1, 6, and 24 h. The levels of IL-8 were measured in cell supernatant by ELISA. Data are mean with SEM of five different normal donors. (* P<0.01; ** P<0.001).

The activation of NF-κB, ERK and AKT pathways induces the expression of pro-inflammatory mediators, such as interleukin-8 (IL-8) [15], [18]. To assess whether CSE affects the release of this cytokine by BMCs, IL-8 concentrations were measured in the culture supernatants. Stimulation of BMCs with CSE elicited a time-dependent increase in IL-8 production. We found that CSE significantly induced IL-8 release at both doses (5% and 10%) (Fig. 6A) and three different time points, 1 h, 6 h and 24 h (Fig. 6B–D). PD98059 and BAY11-7085, but not Wortmannin, reduced the release of IL-8 by CSE-stimulated BMCs. Taken together, these results suggest that ERK1/2 and NF-κB are involved in CSE-mediated IL-8 secretion, whereas AKT is not.

### Effects of CSE on TGF-β1 Release

**Figure 7:**
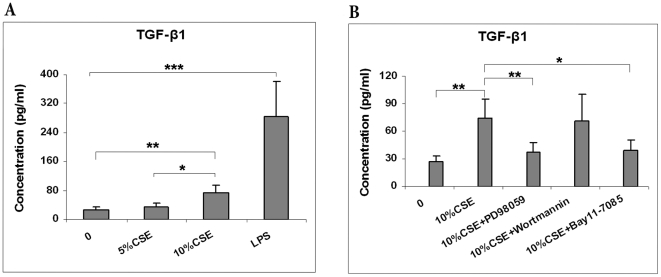
Effects of ERK1/2, AKT and NF-κB signaling pathway on CSE-induced TGF-β1 release. BMCs were pretreated with PD98059 (10 µM), BAY11-7085 (5 µM), or Wortmannin (100 nM) for 60 min, and subsequently were exposed to 10% CSE for 24 h. The levels of TGF-β1 were measured in cell supernatant by ELISA. Data are mean with SEM of five different normal donors. (* P<0.05; ** P<0.01; *** P<0.001).

A large number of in vitro studies have shown that transforming growth factor-β (TGF-β1) plays a critical role in the regulation of hematopoiesis, in particular as a growth inhibitor of progenitor and stem cells [33]. It was found that 10% CSE induced the release of TGF-β1 by BMCs (Fig.7A). Similar to our IL-8 data, inhibition of MAPKs and NF-κB but not PI3K/AKT inhibition prevented the release of TGF-β1, indicating that ERK1/2 and NF-κB are involved in the TGF-β1 release, whereas AKT is not (Fig 7B).

## Discussion

**Figure 8:**
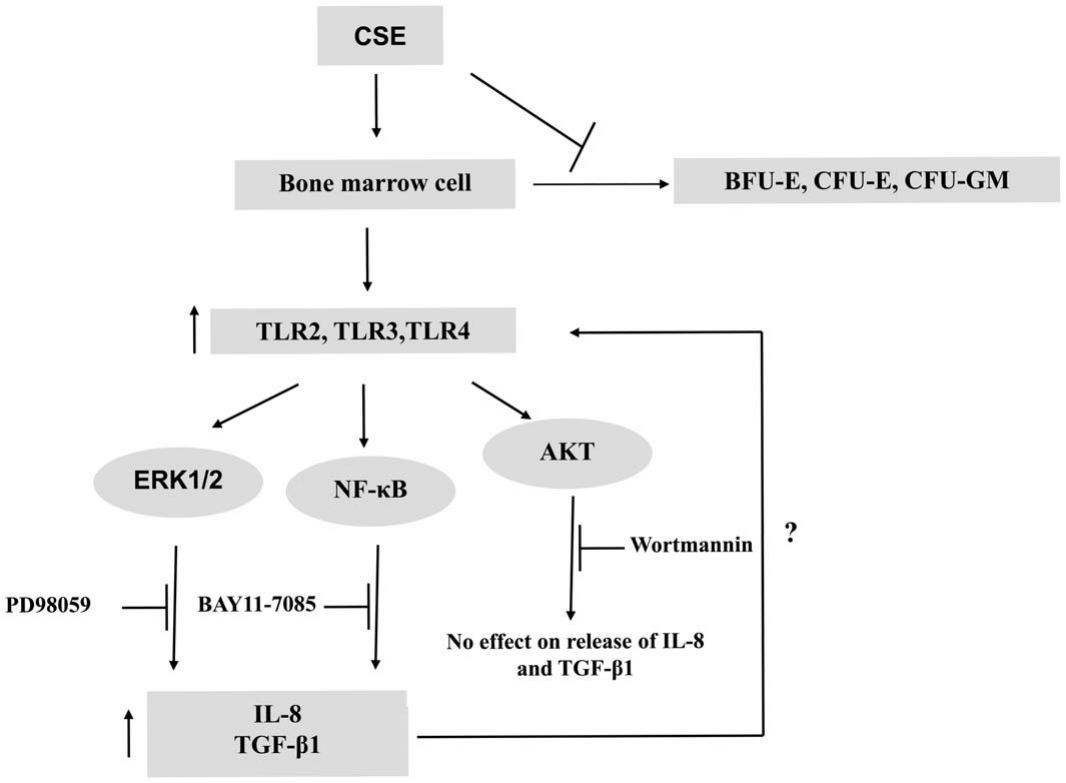
Proposed Model for Overall effects of CSE on BMCs. CSE can inhibit the growth of CFU-E, BFU-E and CFU-GM of human bone marrow cells and induce a significant increase in the expression of TLR2, TLR3 and TLR4. This effect correlates with the activation of ERK1/2 and NF-κB signaling cascades, but not AKT, as demonstrated by specific inhibition of these pathways. The activation of these two pathways by CSE induces the release of TGF-β1 and IL-8, and these cytokines may regulate the expression of Toll receptors. Our hypothesis is that these cytokines and TLR signaling pathways are tightly integrated.

Erythropoiesis and granulopoiesis are multistep processes that involve the differentiation of hemopoietic stem cells into the precursors of mature erythrocytes (BFU-E, CFU-E, erythroid precursors) and monocytes/granulocytes (CFU-GM) respectively [34]–[35]. While these pathways are critical in the development of leukemias and blood cancers, the changes in their phenotype and functionality after direct interaction with CS has not been elucidated. In this study, we examined the effects of CSE on normal human granulocyte-macrophage (CFU-GM) and erythroid (CFU-E, BFU-E) cells. We report that the colony forming ability of these progenitors is highly sensitive to CSE and are affected even at very low doses. In particular, we discovered that the capacity of human BMCs to form BFU-E, CFU-E, and CFU-GM is impaired after treatment with CSE, indicating an *in vitro* ability to inhibit hematopoiesis. This exposure correlated with a significant increase in the expression of the innate immune receptors: TLR2, TLR3 and TLR4, partly mediated through the activation of the ERK1/2 and NF-κB signaling cascades, but not AKT (Figure 8). In addition, our data revealed that CSE modulates the cytokine response of human BMCs by inducing the release of suppressive, tumor differentiating cytokines such as TGF-β1 and IL-8 (normally over-expressed in advanced cancer [36], but not others such as the pro-apoptotic TNF-α, the anti-inflammatory IL-10, or the angiogenic VEGF. The effects observed with our studies suggest that CSE-induced activation of ERK1/2 and NF-κB signaling cascades in BMCs may be a main contributor to abnormal immune responses linked to the pathogenesis of CS. This provides the mechanistic basis to understand the link between cigarette smoking and the deregulation of the bone marrow microenvironment which could lead to leukemia.

TLRs play a key role in pathogen recognition and regulation of immune responses and more recently have become notorious due to their role on the regulation of the tumor microenvironment [37]. This has linked them to chemo-resistance and tumorigenesis processes [38]. However, despite many years of research, mechanisms governing TLR expression as well as their regulation by specific tumorigenic insults remain largely unknown. In this study, we found that exposure of BMCs to 10% CSE increased both the intracellular and extracellular expression of TLR2, TLR3, and TLR4. However, this finding is not surprising due to the known inflammatory nature of CSE. Again, while not completely unexpected, this upregulation of TLRs provide a previously uncharacterized leaving link between leukemias and CSE. Our next step was to try to understand how this up-regulation is induced.

Three main pathways are involved in TLR up-regulation: PI3K/AKT pathway is thought to participate in the TLR2- and TLR4-signaling pathways and can act as either positive or a negative regulators of those cascades; NF-κB signaling has also been implicated in the expression of TLR2 induced by various stimuli, and MAPKs have also been linked to the expression of TLRs by various stimuli [39], [40], [41], [42]. To test the specific role of each one of those pathways we relied on established inhibitors to better understand their role in the induction or TLRs after CSE exposure. Wortmannin is a highly specific and potent inhibitor of PI3K. At nanomolar concentrations this inhibitor irreversibly and fully inhibits PI 3-kinase with very little effect on other signaling molecules (one of the main reasons we chose it in our studies). BAY 11-7085 is an irreversible inhibitor of NF-κB which at higher concentrations can also activate JNK and p38 MAPK, inhibit cell proliferation, and induce apoptosis. PD98059 is a highly selective cell permeable inhibitor of MAP kinase kinase (MEK); it is a noncompetitive inhibitor that affects MEK substrates ATP and ERK. Because CSE is known to activate PI3K/AKT signaling we reasoned that this pathway could be playing a role in the up-regulation of TLRs by BMCs. Instead we found that PI3K/AKT inhibition had no effect on TLR expression but that inhibiting NF-κB effectively suppresses intracellular expression of TLR2 and TLR3 as well as the expression of TLR2 on the surface of the cells, showing that NF-κB is in part responsible for CSE-induced TLR2 and TLR3 expression on BMCs. This pathway has been shown to play a critical role in regulating the inflammatory response by myeloid cells and is known to be potently activated by CS [43], [44]. At the same time, we found that inhibition of NF-κB did not block activation of AKT and ERK1/2 induced by CSE in BMCs. Taken together, CSE activates AKT, ERK1/2 and NF-κB via distinct, non-overlapping mechanisms. Complementing the effects of NF-κB inhibition was ERK which also attenuated intracellular expression of TLR2, suggesting that both of these pathways are activated after exposure of BMC to CSE. Based on the fact that the same exposure induced TGF-β and IL-8 it could be reasoned that activation of ERK and NF-κB may be through the release of these cytokines, which are known to activate these two particular pathways [45]. In addition, hyperactive NF-κB and MAPK pathways have been implicated in tumorigenesis and contribute to the malignant growth of multiple myeloma (MM) [46]. Therefore, our findings here that the activation of these two pathways by CSE furthers the idea that direct exposure to CSE (likely by absorption through the blood during inhalation) can have a profound effect on progenitor cells and the development of abnormal hematopoeisis.

The validity of our system relies on the use of an already established model for the study of cigarette smoke exposure in vitro. Therefore, while this system cannot fully replicate the complex in vivo effects of long-term cigarette smoking, it provides a unique tool to understand the activation of specific pathways after exposure. Furthermore, our study was performed on human primary bone marrow cells, which provide a unique window into how these pathways might also be affected in the population and provides a basis for translational studies. Our future studies will aim at incorporating in vivo animal models to complement the data we have obtained in this study and to better understand the role the bone marrow microenvironment plays on CSE induced toxicities by either acting as a buffer or an accelerant of the tumorigenic process. In summary, we provide the first evidence that CSE affects human bone marrow hematopoiesis and provide new insights into the molecular mechanisms underlying the pathogenesis of bone marrow-related inflammation and cancers in smokers. Moreover, it is plausible that up-regulation of TLRs and activation of inflammatory signaling and production of cytokines by CSE contributes to disease pathogenesis and provides the basis for the use of inhibitors of TLR2, TLR3, ERK1/2 and NF-κB to diminish CS-induced human hematoxic effects as a preventative measure against cancer for long term smokers and their families.

## Materials and Methods

### General Reagents

Rabbit Abs to NF-κB p65, phospho-ERK1/2 (Thr202/Tyr204), phospho-AKT (Ser473), phosphor-IκBα (Ser32), ERK, AKT, PARP, horseradish peroxidase-conjugated anti-rabbit IgG and horseradish peroxidase-conjugated anti-mouse IgG were purchased from Cell Signaling Technology. Mouse anti-β-actin was purchased from Sigma. PD98059, BAY11-7085 and Wortmannin were purchased from Calbiochem (La Jolla, CA). Bone marrow from healthy volunteers was purchased from Cambrex (Gaithersburg, MD), and BMCs were isolated from healthy donors by Ficoll-Hypaque gradient centrifugation. BMCs were maintained in RPMI 1640 supplemented with 10% FBS and 100 U/ml penicillin/streptomycin (control medium).

### Preparation of CSE Solutions

Commercial cigarettes (Marlboro) were smoked continuously by a smoking apparatus, and the mainstream smoke was drawn through 30 ml of serum-free RPMI 1640 medium by application of a vacuum to the vessel containing medium. Each cigarette was smoked for 5 min, and three cigarettes were used per 30 ml of serum-free medium, and the suspension was then adjusted to pH 7.4 and filtered through a 0.22-µm pore filter. This solution was considered to be 100% CSE and was diluted to obtain the desired concentration in each experiment.

### Colony-forming Assay

Methylcellulose colony-forming assays were performed in MethoCult H4434 complete medium with cytokines (StemCell Technologies). In brief, Bone marrow mononuclear cells (BMCs) exposure to 5% CSE, 10% CSE or control for 24 h, then were seeded into complete methylcellulose media, the mixture is placed in 35-mm culture dishes (1×10^5^ cells/each dish) and incubated at 37°C in 5% CO2 for approximately 7–14 days. After incubation, colonies of CFU-E, BFU-E and CFU-GM were indentified and counted using an inverted light microscope. All of the cultures were done in duplicate.

### Enzyme-linked Immunosorbent Assay (ELISA)

BMCs (1×10^6^ cells/ml) were incubated with control medium, 5% CSE, 10% CSE or 1 µg/ml of LPS for 24 h. Supernatants were collected and analyzed for the presence of IL-8, IL-10, TNF-α, TGF-β1, and VEGF by ELISA following the manufacturer's instructions. IL-10, TNF-α and TGF-β1 ELISA kit from eBiosciences, IL-8 ELISA kit from BD Biosciences, and VEGF ELISA kit from R&D Systems.

### Flow Cytometry

BMCs were pretreated with PD98059 (10 µM), BAY11-7085 (5 µM), or Wortmannin (100 nM) for 60 min, and subsequently were exposed to 10% CSE for 24 h. To detect cell surface expression of TLR2, TLR3 and TLR4, the cells were incubated with anti-TLR2-PE, anti-TLR3-PE or anti-TLR4-APC antibodies (ebioscience) for 30 min in the dark on ice, washed and resuspended in PBS. For intracellular TLR2, TLR3 and TLR4 staining, cells were fixed and permeabilized in Cytofix/Cytoperm buffer (BD Bioscience) for 30 min at 4°C before staining for TLR2, TLR3 and TLR4. Flow cytometry was performed using the LSRII (BD Pharmingen), and the results were analyzed using Flowjo 6.3.4 software (TreeStar).

### Nuclear and Cytoplasmic Extracts and Western blot

To study the expression and nuclear translocation of NF-κB, BMCs were incubated with control medium, 5% CSE, 10% CSE, 20% CSE or 1 µg/ml of LPS for 24 h, and then cells were harvested. Nuclear and cytoplasmic protein fractions were extracted using the NE-PER Nuclear and Cytoplasmic Extraction Reagent Kit (Pierce) according to the manufacturer's protocol. The protein concentrations of both nuclear and cytoplasmic extracts were measured by the Bio-Rad protein assay. 30 µg of each cytoplasmic and nuclear extract sample was analyzed by 10% SDS-PAGE and Western blotted using specific antibodies diluted 1∶1000 (NF-κB p65, PARP) or 1∶5000 (β-actin). Anti-mouse HRP or anti-rabbit HRP diluted 1∶2000 was used as the secondary antibody, and detected using an ECL detection system (Amersham Biosciences).

### Western Blot Analysis

BMCs were incubated with control medium, 5% CSE or 10% CSE for 6 h. Cell lysates were prepared, and 30–50 µg of protein was separated on 10% SDS-PAGE and subjected to immunoblotting with antibodies specific for phospho-AKT (Ser-473), phospho-ERK1/2 (Thr202/Tyr204), phosphor-IκBα (Ser32), total AKT, total ERK, and β-actin. In some instances, blots were stripped in stripping buffer (Pierce) at 37°C for 15 min.

### Statistical Analysis

Results are expressed as means ± standard error, Kruskal-Wallis One-way ANOVA and S-N-K were used to examine the significance of the data, and p values <0.05 were considered to be statistically significant.
